# Association between thyroid hormones and insulin resistance indices based on the Korean National Health and Nutrition Examination Survey

**DOI:** 10.1038/s41598-021-01101-z

**Published:** 2021-11-05

**Authors:** Yun Mi Choi, Min Kyung Kim, Mi Kyung Kwak, Dooman Kim, Eun-Gyoung Hong

**Affiliations:** 1 Department of Internal Medicine, Hallym University Dongtan Sacred Heart Hospital, Hallym University College of Medicine, 7 Keunjaebong-gil, Hwaseong, 18450 Korea; 2 Department of Internal Medicine, Hallym University Kangdong Sacred Heart Hospital, Hallym University College of Medicine, Seoul, Korea

**Keywords:** Thyroid diseases, Metabolic syndrome

## Abstract

Thyroid dysfunction has been implicated as a potential pathophysiological factor in glucose homeostasis and insulin resistance (IR). This study aimed to identify the correlation between thyroid dysfunction and IR. We used data from the sixth Korean National Health and Nutrition Examination Survey to evaluate a total of 5727 participants. The triglyceride glucose (TyG) index and homeostasis model assessment of insulin resistance (HOMA-IR) were calculated to represent IR. Correlation analysis was performed between thyroid dysfunction and IR. The log-transformed TSH (LnTSH) and free T4 were significantly correlated with the TyG index (TSH, beta coefficient 0.025, 95% confidence interval [CI] 0.014–0.036, *p* < 0.001; free T4, − 0.110 (− 0.166 to − 0.054), *p* < 0.001) but not HOMA-IR. Overt hypothyroidism is correlated with increased TyG index in pre-menopausal females (0.215 (0.122–0.309) *p* < 0.001). On the other hand, overt hyperthyroidism is correlated with increased HOMA-IR in males (0.304 (0.193–0.416), *p* < 0.001) and post-menopausal females (1.812 (1.717–1.907), *p* < 0.001). In euthyroid subjects, LnTSH and TyG index were significantly correlated in females. In conclusion, both hyperthyroidism and hypothyroidism might be associated with IR but by different mechanisms. It might be helpful to assess IR with appropriate indexes in patients with thyroid dysfunction.

## Introduction

Insulin resistance (IR) is known as a risk factor for hyperglycemia, dyslipidemia, and hypertension, which contribute to the development of atherosclerosis. IR is considered an important and independent contributing factor to cardiovascular disease, a leading cause of morbidity and mortality worldwide^1–3^. Furthermore, several endocrine diseases have IR as a clinical manifestation^4^.

Thyroid hormones are mediators of body metabolism and play an important role in energy homeostasis. Studies on the relationship between thyroid dysfunction and metabolic syndrome, diabetes, and obesity have continued to be increase^5,6^. It has long been recognized that hyperthyroidism promotes hyperglycemia. Diabetic patients with hyperthyroidism have been shown to have poor glycemic control, and thyrotoxicosis have been shown to promote diabetic ketoacidosis in diabetic patients^7,8^. In the case of hypothyroidism, both clinical and subclinical hypothyroidisms have been recognized as risk factors for metabolic syndrome^7,8^. Several studies have investigated the association between thyroid hormone levels and IR. In general, thyroid dysfunction is thought to be related to IR, but the results have been inconsistent^9–12^.

Although the hyperinsulinemic euglycemic clamp (HEC) is the gold standard test for measurement of IR, its practical clinical application is limited for ethical and economic reasons. Therefore, indirect measurement methods such as homeostasis model assessment of insulin resistance (HOMA-IR), Matsuda Index, and quantitative insulin sensitivity check index (QUICKI) have been proposed as surrogate markers for estimating IR^13^. Recently, the triglyceride glucose (TyG) index, calculated from triglycerides and fasting glucose, has been proposed as a reliable and simple surrogate marker of IR in many study fields. Furthermore, this index was not inferior to the HEC or the HOMA-IR for recognizing IR in several human studies^14–16^.

Previous studies reported an association of thyroid function with IR based on the HOMA-IR, a widely used index of IR^17–19^. The relationship between thyroid hormones and the TyG index has not been investigated widely. The aim of this study was to investigate the relationship between thyroid function and IR assessed by the TyG index and HOMA-IR with representative Korean population data.

## Methods

### Data source and study subjects

This study was performed using data from the Korean National Health and Nutrition Examination Survey (KNHANES) VI, which took place from 2013 to 2015 in Korea. It is a nationwide, cross-sectional survey conducted by the Korea Centers for Disease Control and Prevention that uses stratified, multi-stage, clustered probability sampling to select a representative sample of the civilian, non-institutionalized Korean population^20^. Research participants were selected using two-stage stratified cluster sampling of the population and housing census data. The KNHANES obtained written informed consent from every participant prior to completing the survey, and the present study used secondary anonymized data for the analysis. The study protocol was approved by the Institutional Review Board of Hallym University Dongtan Sacred Heart Hospital, Korea (IRB No. 2020-11-010) and was performed in accordance with current guidelines/regulations following the Helsinki Declaration.

Thyroid function testing was conducted in 7061 subjects, one-third of all subjects (n=22,948) aged ≥ 10 years, between 2013 and 2015. The population was selected by stratified subsampling considering the number of inhabitants in each year. Fasting glucose and lipid profile testing were conducted in all subjects aged ≥ 10 years, and fasting insulin was measured only in 2015.

### Laboratory measurements

Blood samples were obtained from each participant’s antecubital vein in the morning after fasting for at least eight hours. As previously reported, serum TSH, free T4, and thyroid peroxidase antibody (TPOAb) were measured with an electrochemiluminescence immunoassay (Cobas8000 E-602/Roche Diagnostics, Mannheim, Germany)^21^. Briefly, TSH was measured with an E-TSH kit (Roche Diagnostics), and the TSH reference interval was determined to be between the 2.5th and 97.5th percentile of the serum TSH levels of the reference population, as previously reported^21^. Serum free T4 was measured using an E-Free T4 kit (Roche Diagnostics), and the reference range was 0.89–1.76 ng/mL. TPOAb was measured using an E-Anti-TPO kit (Roche Diagnos-tics); the normal range for TPOAb in humans is < 34.0 IU/mL.

Lipid profiles were measured with a Hitachi Automatic Analyzer 7600 (Hitachi, Tokyo, Japan) using commercially available kits (Sekisui, Osaka, Japan). Serum total cholesterol and triglycerides were measured by enzymatic methods. Insulin was measured by electrochemiluminescence immunoassay (Cobas 8000/Roche/Germany) using Elecys/insulin (Roche/Germany).

### Classification of thyroid function status and measurement of insulin resistance

As previously defined^21^, the disease-free population was defined as subjects with no prior history of thyroid disease and no history of medication that could influence thyroid function. Participants with past history of dyslipidemia, diabetes mellitus, or a medication history of such drugs were excluded. Personal medical and medication history of dyslipidemia and diabetes mellitus were assessed using a standard questionnaire. Pregnant women and subjects with extremely high triglyceride level (≥ 400 mg/dL) were excluded.

Participants were classified into five groups according to thyroid hormone status as follows: overt hypothyroidism, subclinical hypothyroidism, euthyroid, subclinical hyperthyroidism, and overt hyperthyroidism. Participants with inappropriate thyroid function tests or those in whom we could not rule out secondary hypothyroidism were excluded.

The TyG index was calculated as ln [fasting triglycerides (mg/dL) × fasting glucose (mg/dL)/2]. The HOMA-IR index was calculated using the following formula: HOMA-IR = fasting insulin (μU/mL) × fasting glucose (mg/dL)/405. Because fasting insulin level was measured only in 2015, HOMA-IR could be calculated in a limited population (n = 1881).

### Statistical analysis

Statistical analysis was performed with SAS survey procedures version 9.4 (SAS Institute Inc., Cary, NC, USA). All statistical procedures were conducted to reflect the complex sampling design and sampling weights of KNHANES. Continuous variables are expressed as means ± standard error and are analyzed by ANOVA (Analysis of Variance) test. Categorical variables are presented as numbers and percentages and are analyzed by the Chi-square test. Linear regression analyses were performed to evaluate relationships between thyroid hormone level and IR parameters. Multiple linear regression analyses were performed with adjustment for age, sex, systolic blood pressure (SBP), body mass index (BMI), waist circumference, and alcohol consumption. Regression coefficients were reported as values of β. For TSH, free T4, and HOMA-IR, logarithmic-transformed values were used to normalize the distribution. All *p*-values were two-sided, and values < 0.05 were considered statistically significant.

## Results

### Baseline characteristics of subjects

In total, 5727 subjects were included. Baseline characteristics of subjects according to thyroid dysfunction are shown in Table 1. The mean age of the subjects was 37.99 ± 0.23 years and 47.93% of subjects were female. Though 92.32% of subjects were euthyroid, thyroid dysfunction was more common in females. Height was significantly different between groups, but weight, waist circumference, and BMI did not differ significantly.

**Table 1:** Baseline characteristics of subjects according to thyroid dysfunction.

Variable	Total	Overt hypothyroidism	Subclinical hypothyroidism	Euthyroid	Subclinical hyperthyroidism	Overt hyperthyroidism	*p*-value
**2013–2015**							
Number (%)	5727	30 (0.66)	155 (2.84)	5382 (92.32)	137 (2.64)	23 (0.34)	
Age	37.99 ± 0.23	48.72 ± 2.12	39.70 ± 1.62	37.76 ± 0.24	41.87 ± 1.52	35.31 ± 3.46	< 0.001
Sex (female)	2845 (47.93)	22 (76.66)	101 (65.11)	2639 (47.07)	69 (49.23)	14 (69.97)	< 0.001
Height	165.00 ± 0.13	160.57 ± 1.50	162.05 ± 0.77	165.17 ± 0.14	163.84 ± 0.78	159.89 ± 2.54	< 0.001
Weight	63.82 ± 0.18	63.42 ± 2.16	61.94 ± 1.02	63.92 ± 0.19	63.31 ± 1.14	56.85 ± 2.91	0.05
Waist circumference	79.15 ± 0.18	82.54 ± 1.69	78.96 ± 0.90	79.15 ± 0.18	79.13 ± 0.84	74.42 ± 2.52	0.11
BMI	23.30 ± 0.06	24.55 ± 0.68	23.49 ± 0.29	23.29 ± 0.06	23.43 ± 0.30	22.14 ± 0.86	0.21
Past history of hypertension	488 (9.07)	2 (7.28)	16 (12.01)	455 (8.96)	13 (10.61)	2 (4.27)	0.69
Current medication on hypertension	378 (7.15)	1 (4.43)	13 (9.57)	349 (7.01)	13 (10.61)	2 (4.27)	0.45
Systolic blood pressure	114.23 ± 0.25	116.63 ± 3.62	114.90 ± 1.36	114.15 ± 0.26	115.20 ± 1.53	118.09 ± 4.25	0.76
Fasting glucose	94.51 ± 0.22	97.78 ± 2.79	93.09 ± 0.87	94.52 ± 0.22	94.60 ± 0.96	95.82 ± 3.06	0.40
Triglyceride	114.48 ± 1.05	159.44 ± 18.15	119.32 ± 6.60	114.23 ± 1.06	111.51 ± 6.17	78.83 ± 6.22	< 0.001
TyG index	4.56 ± 0.00	4.75 ± 0.06	4.58 ± 0.03	4.56 ± 0.00	4.55 ± 0.03	4.41 ± 0.04	< 0.001
TPO positivity	316 (5.47)	16 (56.44)	35 (23.77)	246 (4.37)	13 (8.63)	6 (29.76)	< 0.001
**2015**							
Number	1881	19	48	1758	50	6	
Fasting glucose	94.75 ± 0.38	96.60 ± 3.90	90.89 ± 1.41	94.83 ± 0.39	94.99 ± 1.40	96.49 ± 6.78	0.09
Triglyceride	115.27 ± 1.75	170.86 ± 24.65	116.77 ± 13.26	114.74 ± 1.71	112.63 ± 11.00	70.12 ± 8.84	< 0.001
HOMA-IR	2.12 ± 0.05	2.40 ± 0.36	2.10 ± 0.24	2.12 ± 0.06	2.11 ± 0.46	2.77 ± 1.00	0.91

*BMI* body mass index, *TyG index* triglyceride glucose index, *TPO* thyroid peroxidase antibody, *HOMA_IR* homeostasis model assessment of insulin resistance.

### Association between thyroid function and TyG index

We evaluated the association between TSH and TyG index. Log-transformed TSH (LnTSH) was positively associated with TyG index after adjustment for age, sex, SBP, BMI, waist circumference, and alcohol consumption (beta coefficient 0.025, 95% confidence interval [CI] 0.014–0.036, *p *< 0.001) (Table 2). The associations were more prominent in female patients (Male, beta coefficient 0.016, 95% CI − 0.008 to 0.000, *p *= 0.032; Female, beta coefficient 0.035, 95% CI 0.021–0.048, *p *< 0.001). On the other hand, log-transformed free T4 [Ln(free T4)] exhibited a negative association with the TyG index (beta coefficient − 0.110, 95% CI − 0.166 to − 0.054, *p *< 0.001).

**Table 2:** Association between thyroid function and TyG index.

Model^#^	Variable	Total (n = 5727)				Male (n = 2882)				Female (n = 2845)			
		Beta	95% CI		P value	Beta	95% CI		P value	Beta	95% CI		P value
1	LnTSH	0.018	0.005	0.030	0.005*	0.002	− 0.016	0.020	0.831	0.041	0.025	0.056	< 0.0001*
2	LnTSH	0.025	0.014	0.036	< 0.0001*	0.016	0.008	0.000	0.032*	0.035	0.021	0.048	< 0.0001*
3	LnTSH	0.025	0.014	0.035	< 0.0001*	0.016	0.000	0.032	0.049*	0.034	0.021	0.047	< 0.0001*
1	Ln(free T4)	− 0.131	− 0.190	− 0.073	< 0.0001*	− 0.295	− 0.386	− 0.205	< 0.0001*	− 0.248	− 0.327	− 0.168	< 0.0001*
2	Ln(free T4)	− 0.110	− 0.166	− 0.054	< 0.0001*	− 0.086	− 0.176	0.003	0.059	− 0.135	− 0.207	− 0.062	< 0.0001*
3	Ln(free T4)	− 0.113	− 0.168	− 0.058	< 0.0001*	− 0.102	− 0.191	− 0.014	0.024*	− 0.129	− 0.200	− 0.059	< 0.0001*

^#^Model 1: linear regression analysis, 2: multiple regression analysis after adjustment for age, sex, systolic blood pressure, BMI, alcohol consumption, 3: multiple regression analysis after adjustment for age, sex, systolic blood pressure, waist circumference, alcohol consumption *P-values < 0.05 were considered as statistically significant.

When analysis was performed according to five categorical thyroid functions, the same trend was shown (Table 3). Overt hypothyroidism was positively correlated with the TyG index (beta coefficient 0.127, 95% CI 0.036–0.217, *p *= 0.006), while overt hyperthyroidism showed a negative correlation (beta coefficient − 0.093, 95% CI − 0.159 to − 0.027, *p *= 0.006). Subclinical dysfunction did not exhibit significant associations in all subjects. However, subgroup analysis showed that the association was only statistically significant in females with hypothyroidism. In female patients, association is evident with subclinical thyroid dysfunction.

**Table 3:** Association between categorical thyroid function and TyG index.

Model^#^	Variable	Total (n = 5727)				Male (n = 2882)				Female (n = 2845)			
		Beta	95% CI		P value	Beta	95% CI		P value	Beta	95% CI		P value
1					< 0.0001*				0.004*				< 0.0001*
	Overt hypothyroidism	0.194	0.081	0.308	0.001	0.156	− 0.092	0.404	0.216	0.263	0.136	0.391	< 0.0001
	Subclinical hypothyroidism	0.024	− 0.028	0.076	0.365	− 0.034	− 0.120	0.051	0.427	0.096	0.033	0.159	0.003
	Euthyroid	0 (ref)				0 (ref)				0 (ref)			
	Subclinical hyperthyroidism	− 0.008	− 0.063	0.047	0.788	0.046	− 0.029	0.121	0.231	− 0.056	− 0.127	0.015	0.120
	Overt hyperthyroidism	− 0.147	− 0.232	− 0.062	0.001	− 0.223	− 0.351	− 0.095	0.001	− 0.066	− 0.175	0.043	0.234
2					< 0.0001*				0.238				< 0.0001*
	Overt hypothyroidism	0.127	0.036	0.217	0.006	0.012	− 0.220	0.244	0.919	0.166	0.075	0.257	< 0.0001
	Subclinical hypothyroidism	0.048	− 0.003	0.100	0.066	0.018	− 0.077	0.112	0.715	0.067	0.010	0.125	0.022
	Euthyroid	0 (ref)				0 (ref)				0 (ref)			
	Subclinical hyperthyroidism	− 0.024	− 0.072	0.024	0.330	− 0.011	− 0.078	0.056	0.748	− 0.043	− 0.110	0.023	0.201
	Overt hyperthyroidism	− 0.093	− 0.159	− 0.027	0.006	− 0.151	− 0.280	− 0.022	0.022	− 0.071	− 0.145	0.003	0.060
3					0.001*				0.320				< 0.0001*
	Overt hypothyroidism	0.121	0.031	0.211	0.009	0.006	− 0.233	0.245	0.961	0.160	0.073	0.247	< 0.0001
	Subclinical hypothyroidism	0.049	− 0.003	0.101	0.064	0.009	− 0.088	0.107	0.849	0.071	0.015	0.127	0.013
	Euthyroid	0 (ref)				0 (ref)				0 (ref)			
	Subclinical hyperthyroidism	− 0.018	− 0.063	0.028	0.442	− 0.002	− 0.067	0.064	0.958	− 0.039	− 0.101	0.023	0.220
	Overt hyperthyroidism	− 0.086	− 0.152	− 0.020	0.011	− 0.159	− 0.304	− 0.014	0.032	− 0.059	− 0.124	0.006	0.077

^#^Model 1: linear regression analysis, 2: multiple regression analysis after adjustment for age, sex, systolic blood pressure, BMI, alcohol consumption, 3: multiple regression analysis after adjustment for age, sex, systolic blood pressure, waist circumference, alcohol consumption *P-values < 0.05 were considered as statistically significant.

### Association between thyroid function and HOMA-IR index

We evaluated the association between TSH and HOMA-IR index. TSH and free T4 were not significantly correlated with HOMA-IR (Table 4). There was also no correlation in subgroup analysis by sex.

**Table 4:** Association between thyroid function and HOMA-IR index.

Model^#^	Variable	Total (n = 1881)				Male (n = 949)				Female (n = 932)			
		Beta	95% CI		P value	Beta	95% CI		P value	Beta	95% CI		P value
1	LnTSH	0.023	− 0.022	0.068	0.321	0.031	− 0.037	0.099	0.376	0.021	− 0.038	0.081	0.483
2	LnTSH	0.000	− 0.037	0.037	0.998	− 0.015	− 0.070	0.041	0.609	0.018	− 0.033	0.068	0.498
3	LnTSH	0.003	− 0.033	0.040	0.851	− 0.004	− 0.059	0.051	0.893	0.014	− 0.036	0.064	0.580
1	Ln(free T4)	0.083	− 0.140	0.307	0.465	− 0.021	− 0.385	0.342	0.909	0.047	− 0.278	0.372	0.777
2	Ln(free T4)	− 0.054	− 0.308	0.201	0.680	− 0.052	− 0.423	0.319	0.782	− 0.018	− 0.348	0.313	0.917
3	Ln(free T4)	− 0.075	− 0.328	0.179	0.563	− 0.142	− 0.503	0.219	0.441	0.004	− 0.323	0.332	0.979

^#^Model 1: linear regression analysis, 2: multiple regression analysis after adjustment for age, sex, systolic blood pressure, BMI, alcohol consumption, 3: multiple regression analysis after adjustment for age, sex, systolic blood pressure, waist circumference, alcohol consumption; TSH, free T4, and HOMA-IR are logarithmic-transformed. *P-values < 0.05 were considered as statistically significant.

When analysis was performed according to five categorical thyroid functions, only male subjects with overt hyperthyroidism exhibited a strong positive correlation with the HOMA-IR index (beta coefficient 0.304, 95% CI 0.193–0.416, *p *< 0.001) (Table 5).

**Table 5:** Association between categorical thyroid function and HOMA-IR index.

Model^#^	Variable	Total (n = 1881)				Male (n = 949)				Female (n = 932)			
		Beta	95% CI		P value	Beta	95% CI		P value	Beta	95% CI		P value
1					0.365				< 0.0001*				0.348
	Overt hypothyroidism	0.210	− 0.101	0.521	0.184	0.293	− 0.369	0.955	0.386	0.234	− 0.113	0.580	0.186
	Subclinical hypothyroidism	0.027	− 0.160	0.214	0.776	− 0.128	− 0.383	0.127	0.326	0.176	− 0.109	0.461	0.226
	Euthyroid	0 (ref)				0 (ref)				0 (ref)			
	Subclinical hyperthyroidism	− 0.147	− 0.368	0.075	0.194	− 0.158	− 0.423	0.107	0.243	− 0.148	− 0.510	0.214	0.422
	Overt hyperthyroidism	0.245	− 0.315	0.805	0.391	0.846	0.794	0.897	< 0.0001	0.218	− 0.368	0.804	0.466
2					0.240				< 0.0001*				0.234
	Overt hypothyroidism	0.284	0.008	0.560	0.044	0.496	− 0.158	1.150	0.137	0.241	− 0.064	0.545	0.121
	Subclinical hypothyroidism	− 0.055	− 0.210	0.101	0.490	− 0.165	− 0.378	0.047	0.127	0.037	− 0.170	0.243	0.728
	Euthyroidism	0 (ref)				0 (ref)				0 (ref)			
	Subclinical hyperthyroidism	− 0.048	− 0.221	0.124	0.582	0.006	− 0.210	0.222	0.958	− 0.175	− 0.399	0.050	0.127
	Overt hyperthyroidism	0.254	− 0.317	0.824	0.383	0.304	0.193	0.416	< 0.0001	0.255	− 0.377	0.887	0.429
3					0.139				< 0.0001*				0.211
	Overt hypothyroidism	0.263	0.038	0.488	0.022	0.496	− 0.100	1.092	0.103	0.209	− 0.025	0.444	0.080
	Subclinical hypothyroidism	− 0.044	− 0.194	0.106	0.566	− 0.184	− 0.363	− 0.004	0.045	0.069	− 0.148	0.286	0.533
	Euthyroidism	0 (ref)				0 (ref)				0 (ref)			
	Subclinical hyperthyroidism	− 0.032	− 0.202	0.138	0.711	0.020	− 0.189	0.229	0.850	− 0.146	− 0.391	0.099	0.241
	Overt hyperthyroidism	0.306	− 0.235	0.846	0.267	0.255	0.138	0.372	< 0.0001	0.315	− 0.288	0.917	0.306

^#^Model 1: linear regression analysis, 2: multiple regression analysis after adjustment for age, sex, systolic blood pressure, BMI, alcohol consumption, 3: multiple regression analysis after adjustment for age, sex, systolic blood pressure, waist circumference, alcohol consumption; HOMA-IR is logarithmic-transformed. *P-values < 0.05 were considered as statistically significant.

### Subgroup analysis according to menopausal status in female

We performed subgroup analysis in female patients according to menopausal status. Correlation between thyroid function and TyG index were only significant in premenopausal women (LnTSH, beta coefficient 0.044, 95% CI 0.029–0.058, *p *< 0.001; Overt hypothyroidism, beta coefficient 0.215, 95% CI 0.122–0.309, *p *< 0.001) (Supplementary Tables S1, S2). The association between numeric TSH and HOMA-IR was positively correlated in premenopausal females, although the significance was marginal (Supplementary Table S3). Overt hypothyroidism and HOMA-IR had significant positive correlations in premenopausal females (beta coefficient 0.336, 95% CI 0.058–0.614, *p *= 0.018). On the other hand, overt hyperthyroidism and HOMA-IR had significant positive correlations in postmenopausal females (beta coefficient 1.812, 95% CI 1.717–1.907, *p *< 0.001) (Supplementary Table S4).

### Subgroup analysis according to TPO positivity

There was no significant difference according to TPO positivity (data not shown).

### Association between TSH and insulin resistance indexes in euthyroid subjects

Additionally, we performed subgroup analysis only in subjects who were euthyroid. The TSH and TyG index were significantly correlated in female subjects (beta coefficient 0.045, 95% CI 0.023–0.067, *p *< 0.001). On the other hand, there was no correlation between TSH and HOMA-IR (beta coefficient − 0.002, 95% CI − 0.063 to 0.059, *p *= 0.955) (Supplementary Table S5).

## Discussion

In this study, we investigated the relationship between thyroid function and IR using data from the KNHANES VI, and showed that overt hypothyroidism is correlated with increased IR represented by the TyG index in pre-menopausal females. On the other hand, overt hyperthyroidism is correlated with increased IR, as represented by the HOMA-IR in males and post-menopausal females. LnTSH was significantly correlated with the TyG index not only in whole subjects, but also in euthyroid subjects.

IR is a representative characteristic of obesity, diabetes, and metabolic syndrome and is associated with cardiovascular disease^13^. Therefore evaluation of IR is very important in a number of clinical states where insulin sensitivity is compromised. The HEC is the gold standard method for determination of insulin sensitivity^22^, but this method is invasive, laborious, and expensive, and it can only be used for research purposes. Therefore, a number of surrogate markers have been developed and improved to simply measure IR^13,23^.

Previous studies reported an increased HOMA-IR index and decreased Matsuda index in hypothyroidism and hyperthyroidism^17–19,24^. On the other hand, short lasting hypothyroidism induced by thyroid hormone withdrawal in thyroidectomized patients did not show any correlation with HOMA-IR index^25^. Some other studies analyzed correlations between thyroid function and IR in euthyroid subjects, but the results are inconsistent. In a recent Korean study, free T3 was positively correlated with HOMA-IR^26^, whereas another study showed an inverse correlation between free T4 and HOMA-IR^27^. Another study, also using KNHANES VI data from children and adolescents, could not show any correlation between thyroid hormone level and IR^28^. This inconsistency might originate from the difference in thyroid hormone action depending on the organs responsible for IR. First thyroid hormones interfere insulin action and stimulate hepatic gluconeogenesis and glycogenolysis. On the other hand, glucose transport and glycolysis are also increased by upregulated gene expression of glucose transporter type-4 (GLUT-4) and phosphoglycerate kinase by thyroid hormones. Therefore, they can facilitate glucose disposal and utilization in peripheral tissues by acting synergistically with insulin^29–33^. Furthermore, thyroid dysfunction has been known to have profound effects on body composition. Previously, several studies reported positive association between serum TSH levels and BMI^34^. Another study observed positive correlation between free T3 and parameters of adiposity and negative correlation between free T3 and parameters of muscularity^26,35^. Not only do thyroid hormones directly affect IR, but changes in body composition associated with thyroid dysfunction might also indirectly affect insulin resistance.

Our result can be explained by the different aspects of IR reflected by the two indices. The TyG index is a better indicator of peripheral IR as it mainly reflects the IR in the muscle. In contrast, HOMA-IR reflects IR mainly in the liver and is a better indicator for hepatic IR. Therefore, these differences can be observed^36–38^. TyG index should be interpreted cautiously in hyperthyroid status which lowers triglyceride levels but has been traditionally known to be associated to IR. In addition, our study showed that IR changes according to sex or menopausal status, suggesting that there might be crosstalk between estrogen and thyroid hormone in the development of IR.

This study had some limitations. First, thyroid hormones and IR indexes were only measured at one point, meaning that the effects of thyroid function changes on IR could not be determined. Second, this is a retrospective and cross‐sectional study that cannot explain causality. Third, this study was performed in a single ethnicity and cannot be generalized to diverse ethnic groups.

The results of the present study showed that there is a significant association between the IR and thyroid hormones. Despite the aforementioned limitations, this study is of clinical significance as it is the first study to examine the association between TyG index and thyroid hormones (both hyperthyroidism and hypothyroidism). Assessment of IR is essential in any clinical situations that could compromise insulin sensitivity, and it should be managed aggressively to reduce the impending risk. Thyroid dysfunction is one of the most common endocrine disorders and both hyperthyroidism and hypothyroidism might be associated with IR by different mechanisms. It might be helpful to assess IR with appropriate indexes in thyroid dysfunction.

## Supplementary Information

Supplementary Tables.

## Data Availability

Data are available from the Korea National Health and Nutrition Examination Survey (KNHANES) website (http://knhanes.cdc.go.kr).
