# Supplementary material for: Association between thyroid hormones and insulin resistance indices based on the Korean National Health and Nutrition Examination Survey
Source: Sci Rep. 2021 Nov 5;11:21738. doi: 10.1038/s41598-021-01101-z (PMC8571382; doi:10.1038/s41598-021-01101-z)
Supplement: Supplementary file 1 — Supplementary Tables. [file 41598_2021_1101_MOESM1_ESM.pdf]

Supplementary Table S1. Subgroup analysis of the association between thyroid function and TyG index according to menopausal status

		Pre-menopausal (n=2200)				Post-menopausal (n=645)			
Model#	variable	Beta	95% CI		P value	Beta	95% CI		P value
1	LnTSH	0.046	0.027	0.065	<.0001*	0.013	-0.013	0.040	0.328
2	LnTSH	0.044	0.029	0.058	<.0001*	0.011	-0.016	0.037	0.426
3	LnTSH	0.042	0.028	0.057	<.0001*	0.013	-0.012	0.038	0.319
1	Ln(free T4)	-0.263	-0.366	-0.160	<.0001*	-0.128	-0.247	-0.009	0.035*
2	Ln(free T4)	-0.145	-0.236	-0.055	0.002*	-0.122	-0.240	-0.003	0.044*
3	Ln(free T4)	-0.129	-0.218	-0.040	0.005*	-0.133	-0.247	-0.020	0.022*

#Model 1: linear regression analysis, 2: multiple regression analysis after adjustment for age, sex, systolic blood pressure, BMI, alcohol consumption, 3: multiple regression analysis after adjustment for age, sex, systolic blood pressure, waist circumference, alcohol consumption. **P*-values <0.05 were considered as statistically significant

Supplementary Table S2. Subgroup analysis of the association between categorical thyroid function and TyG index according to menopausal status

		Pre-menopausal (n=2200)				Post-menopausal (n=645)			
Model#	variable	Beta	95% CI		P value	Beta	95% CI		P value
1					<.0001*				0.550
	Overt hypothyroidism	0.342	0.192	0.492	<.0001	0.046	-0.137	0.229	0.621
	Subclinical hypothyroidism	0.091	0.014	0.169	0.021	0.054	-0.050	0.158	0.306
	Euthyroid	0 (ref)				0 (ref)			
	Subclinical hyperthyroidism	-0.084	-0.156	-0.012	0.023	0.027	-0.125	0.179	0.729
	Overt hyperthyroidism	-0.062	-0.184	0.061	0.323	-0.110	-0.288	0.067	0.222
2					<.0001*				0.383
	Overt hypothyroidism	0.215	0.122	0.309	<.0001	0.048	-0.087	0.184	0.484
	Subclinical hypothyroidism	0.058	-0.011	0.127	0.100	0.071	-0.032	0.173	0.178
	Euthyroid	0 (ref)				0 (ref)			
	Subclinical hyperthyroidism	-0.074	-0.148	0.001	0.052	0.058	-0.085	0.200	0.427
	Overt hyperthyroidism	-0.071	-0.144	0.003	0.058	-0.101	-0.284	0.083	0.282
3					<.0001*				0.268
	Overt hypothyroidism	0.204	0.107	0.300	<.0001	0.053	-0.071	0.177	0.406
	Subclinical hypothyroidism	0.061	-0.008	0.131	0.084	0.072	-0.022	0.166	0.134
	Euthyroid	0 (ref)				0 (ref)			
	Subclinical hyperthyroidism	-0.069	-0.138	-0.001	0.048	0.059	-0.078	0.196	0.399
	Overt hyperthyroidism	-0.056	-0.121	0.009	0.089	-0.099	-0.261	0.064	0.233

#Model 1: linear regression analysis, 2: multiple regression analysis after adjustment for age, sex, systolic blood pressure, BMI, alcohol consumption, 3: multiple regression analysis after adjustment for age, sex, systolic blood pressure, waist circumference, alcohol consumption. **P*-values <0.05 were considered as statistically significant

Supplementary Table S3. Subgroup analysis of the association between thyroid function and HOMA-IR index according to menopausal status

		Pre-menopausal (n=728)				Post-menopausal (n=204)			
Model#	variable	Beta	95% CI		P value	Beta	95% CI		P value
1	LnTSH	0.058	-0.001	0.117	0.052	-0.071	-0.194	0.051	0.253
2	LnTSH	0.052	0.001	0.103	0.046*	-0.068	-0.170	0.035	0.196
3	LnTSH	0.046	-0.003	0.095	0.067	-0.066	-0.167	0.035	0.201
1	Ln(free T4)	-0.226	-0.547	0.094	0.167	0.635	0.118	1.152	0.016*
2	Ln(free T4)	-0.328	-0.671	0.014	0.060	0.448	-0.083	0.980	0.098
3	Ln(free T4)	-0.269	-0.591	0.052	0.100	0.389	-0.180	0.958	0.180

#Model 1: linear regression analysis, 2: multiple regression analysis after adjustment for age, sex, systolic blood pressure, BMI, alcohol consumption, 3: multiple regression analysis after adjustment for age, sex, systolic blood pressure, waist circumference, alcohol consumption. **P*-values <0.05 were considered as statistically significant

Supplementary Table S4. Subgroup analysis of the association between categorical thyroid function and HOMA-IR index according to menopausal status

		Pre-menopausal (n=728)				Post-menopausal (n=204)			
Model#	variable	Beta	95% CI		P value	Beta	95% CI		P value
1					<.0001*				<.0001*
	Overt hypothyroidism	0.418	0.251	0.585	<.0001	-0.116	-0.790	0.559	0.737
	Subclinical hypothyroidism	0.267	-0.062	0.595	0.111	-0.108	-0.581	0.365	0.655
	Euthyroid	0 (ref)				0 (ref)			
	Subclinical hyperthyroidism	-0.187	-0.623	0.248	0.399	-0.033	-0.446	0.380	0.876
	Overt hyperthyroidism	-0.093	-0.363	0.176	0.497	1.812	1.717	1.907	<.0001
2					0.042*				<.0001*
	Overt hypothyroidism	0.374	-0.016	0.763	0.060	0.106	-0.275	0.486	0.585
	Subclinical hypothyroidism	0.137	-0.060	0.334	0.171	-0.241	-0.610	0.128	0.200
	Euthyroid	0 (ref)				0 (ref)			
	Subclinical hyperthyroidism	-0.279	-0.535	-0.024	0.032	0.180	-0.169	0.530	0.312
	Overt hyperthyroidism	-0.016	-0.400	0.369	0.936	1.812	1.677	1.946	<.0001
3					0.034 *				<.0001*
	Overt hypothyroidism	0.336	0.058	0.614	0.018	0.069	-0.319	0.457	0.728
	Subclinical hypothyroidism	0.158	-0.062	0.377	0.158	-0.163	-0.533	0.207	0.386
	Euthyroid	0 (ref)				0 (ref)			
	Subclinical hyperthyroidism	-0.255	-0.535	0.025	0.075	0.207	-0.014	0.427	0.066
	Overt hyperthyroidism	0.053	-0.296	0.402	0.765	1.827	1.690	1.965	<.0001

#Model 1: linear regression analysis, 2: multiple regression analysis after adjustment for age, sex, systolic blood pressure, BMI, alcohol consumption, 3: multiple regression analysis after adjustment for age, sex, systolic blood pressure, waist circumference, alcohol consumption *P-values <0.05 were considered as statistically significant

Supplementary Table S5. Subgroup analysis of the association between categorical thyroid function and TyG index/HOMA-IR index in euthyroid population

			Total (n=5315)				Male (n=2701)				Female (n=2614)			
Outcome	Model#	variable	Beta	95% CI		P value	Beta	95% CI		P value	Beta	95% CI		P value
TyG index	1	LnTSH	0.018	-0.001	0.037	0.058	0.006	-0.019	0.032	0.629	0.052	0.028	0.077	<.0001*
	2	LnTSH	0.030	0.014	0.046	<.0001*	0.020	-0.002	0.042	0.077	0.045	0.023	0.067	<.0001*
			Total (n=1741)				Male (n=886)				Female (n=855)			
HOMA-IR	1	LnTSH	0.018	-0.053	0.089	0.611	0.051	-0.070	0.173	0.406	-0.009	-0.090	0.072	0.822
	2	LnTSH	-0.002	-0.063	0.059	0.955	-0.015	-0.116	0.087	0.775	0.010	-0.056	0.077	0.762

#Model 1: linear regression analysis, 2: multiple regression analysis after adjustment for age, sex, systolic blood pressure, BMI, alcohol consumption. **P*-values <0.05 were considered as statistically significant
